# Circulating Irisin Levels Are Not Regulated by Nutritional Status, Obesity, or Leptin Levels in Rodents

**DOI:** 10.1155/2015/620919

**Published:** 2015-10-19

**Authors:** Mar Quiñones, Cintia Folgueira, Estrella Sánchez-Rebordelo, Omar Al-Massadi

**Affiliations:** ^1^Department of Physiology, CIMUS, University of Santiago de Compostela, Sanitary Research Institute of Santiago de Compostela (IDIS), 15782 Santiago de Compostela, Spain; ^2^CIBER Physiopathology of Obesity and Nutrition (CIBERobn), 15706 Santiago de Compostela, Spain; ^3^Endocrine Physiopathology Group, Sanitary Research Institute of Santiago de Compostela (IDIS), University Hospital Complex of Santiago (CHUS/SERGAS), 15706 Santiago de Compostela, Spain

## Abstract

Irisin is a cleaved and secreted fragment of fibronectin type III domain containing 5 (FNDC5) that is mainly released by skeletal muscle and was proposed to mediate the beneficial effects of exercise on metabolism. In the present study we aim to investigate the regulation of the circulating levels of irisin in obese animal models (diet-induced obese (DIO) rats and leptin-deficient (ob/ob) mice), as well as the influence of nutritional status and leptin. Irisin levels were measured by Enzyme-Linked Immunosorbent Assay (ELISA) and Radioimmunoassay (RIA). Serum irisin levels remained unaltered in DIO rats and ob/ob mice. Moreover, its circulating levels were also unaffected by fasting, leptin deficiency, and exogenous leptin administration in rodents. In spite of these negative results we find a negative correlation between irisin and insulin in DIO animals and a positive correlation between irisin and glucose under short-term changes in nutritional status. Our findings indicate that serum irisin levels are not modulated by different physiological settings associated to alterations in energy homeostasis. These results suggest that in rodents circulating levels of irisin are not involved in the pathophysiology of obesity and could be unrelated to metabolic status; however, further studies should clarify its precise role in states of glucose homeostasis imbalance.

## 1. Introduction

The combination of energy-dense diets and sedentary lifestyles has accelerated the incidence of obesity and its comorbidities including dyslipidemia, diabetes mellitus, and cardiovascular disease. Inflammatory processes play a crucial role in the development of obesity. Adipose tissue release cytokines such as IL-6 and IL-1*β* in obesity that targets several tissues such as the heart, the pancreas, or the liver [1]. Physical inactivity causes accumulation of visceral fat that induces systemic low grade inflammation and in turn exercise promotes a variety of metabolically beneficial effects in the organism [2]. However, its mechanism of action remains elusive and many efforts are focused to characterize new molecular targets or mediators of these healthy benefits. The skeletal muscle is considered nowadays as a complete endocrine organ [3] and there are more than 1000 genes activated by exercise [4]. One of these proposed mediators is the peroxisome proliferator-activated receptor gamma coactivator 1 alpha (PGC1-*α*), a transcriptional coactivator released by the muscle that induces mitochondrial biogenesis and thus thermogenesis [5, 6] as well as modulating glucose, lipid, and energy homeostasis [7, 8]. PGC1-*α* induces the expression of the fibronectin type III domain containing 5 (FNDC5) gene that is cleaved and secretes a new hormone from skeletal muscle named irisin [8]. FNDC5 was simultaneously characterized in 2002 by two independent groups [9, 10], but it was ten years later when irisin attracted more interest because it was reported to act as a mediator of the beneficial effect of exercise [8], increasing metabolic uncoupling and caloric expenditure and promoting browning [8, 11, 12], resulting in an improvement of obesity and glucose homeostasis. However, further studies performed in animals and humans reported controversial results and the physiological role of irisin is still under debate [2, 8, 13–18].

On the other hand, several studies assessed circulating levels of irisin in humans and found controversial results because some of them reported an increase of irisin under conditions of obesity [19, 20] while others found a negative association between irisin levels and obesity [21]. Furthermore recently no association was found between these two parameters in humans [22]. Accordingly, circulating irisin levels decreased after surgical [23] or dietary-induced [24] weight loss. In rodents, gene expression results showed that skeletal muscle FNDC5 mRNA levels were proportional to circulating leptin levels [25] and the regulation of FNDC5 expression in DIO animals was different depending on the fat depot [26]. However, there are no data on circulating irisin levels in obese rodents and its regulation under certain physiological conditions associated to body weight changes is very scarce. The aim of this study was to assess the regulation of serum irisin levels by nutritional status, leptin, and diet-induced obesity in rodents.

## 2. Material and Methods

### 2.1. Animals

Male Sprague-Dawley rats (bred in the Animalario Xeral of USC; Santiago de Compostela, Spain) and ob/ob mice (Charles River, Barcelona, Spain) were housed under conditions of controlled illumination (12 : 12-h light/dark cycle), humidity, and temperature. Animals were fed with a standard diet (Scientific Animal Food & Engineering, proteins 16%, carbohydrates 60%, and fat 3%) and tap water* ad libitum* unless otherwise indicated. The animals were sacrificed in a room separate from other experimental animals, and blood samples were centrifuged at 1500 g for 10 min and the serum was harvested and stored at −80°C until an analysis could be performed. All experimental procedures were reviewed and approved by the Ethics Committees of the University of Santiago de Compostela in accordance with the institutional guidelines and in strict compliance with the European Union normative for the care and use of experimental animals. The number of animals used in each experimental setting is indicated in the corresponding figure's caption.

### 2.2. Experimental Setting 1: Effect of the Diet on Serum Irisin Levels

After weaning, 6-week-old male Sprague-Dawley rats were either fed a high-fat (HF) diet (60% by energy) or a low-fat (LF) diet (10% by energy) (reference #: D12492 and D12450B resp., Research Diets, NJ, US) for 10 weeks.

### 2.3. Experimental Setting 2: Effects of Food Deprivation on Serum Irisin Levels

10-week-old male rats were deprived of food for 48 h, or refed for 24 h after fasting while the control group was fed* ad libitum* [27, 28]. All animals had free access to tap water.

### 2.4. Experimental Setting 3: Effects of Leptin on Serum Irisin Levels

The effects of systemic leptin administration on serum irisin levels were studied in leptin deficient mice (ob/ob mice). Leptin-deficient animals were referred to wild type (WT) control animals and were distributed in three groups: (a) i.p. vehicle fed* ad libitum*, (b) i.p. vehicle after 36 h fasting, and (c) i.p. leptin in fed* ad libitum* mice. Animals were treated with recombinant leptin (L-4146, Sigma-Aldrich) at a dose of 0.5 mg/kg of body weight every 6 h for 3 days (intraperitoneal injection) [29].

### 2.5. Measurement of Body Composition

Body composition (fat and lean mass) was assessed using Nuclear Magnetic Resonance imaging system (Whole Body Composition Analyzer; EchoMRI, Houston, USA) as previously shown [30, 31].

### 2.6. Measurement of Biochemical and Hormonal Parameters

Irisin and insulin levels were determined by ELISA using reagents kits and methods provided by Phoenix Pharmaceuticals Inc. and by Millipore corporation, respectively. These kits are suitable for human, rats, and mice and were used in previous studies [20, 24, 32–34]. The quantitative measurement of irisin in serum samples was performed using a commercial ELISA kit directed against amino acids 33–142 of the FNDC5 protein (Irisin ELISA Kit EK-067-52; Phoenix Pharmaceuticals Inc., CA) according to the manufacturer's instructions. The absorbance from each sample was measured in duplicate using a spectrophotometric microplate reader at wavelength of 450 nm (Versamax Microplate Reader; Associates of Cape Cod Incorporated, East Falmouth, MA). The intra- and interassay coefficients of variation for the kit were 4–6% and 8–10%, respectively. Irisin levels were determined also by RIA using reagents kits and methods provided by Phoenix Pharmaceuticals Inc. (Belmont, CA; Cat. No. RK-067-16). For testing serum irisin levels, the samples were obtained from trunk blood by decapitation and were collected in tubes for serum separation (BD Vacutainer SST II Advance). Results were expressed as ng per milliliter of irisin in serum. Serum samples were tested in duplicate within one assay, and the results were expressed in terms of the rat irisin standard (ng/mL).

The absorbance from each sample with regard to insulin levels was measured in duplicate using a spectrophotometric microplate reader at wavelength of 450 nm and 590 nm. The intra- and interassay coefficients of variation for the kit were 1–4% and 6–9%, respectively. Serum samples were tested in duplicate within one assay, and the results were expressed in terms of the rat insulin standard (ng/mL).

Glucose and total cholesterol was determined using colorimetric assays (Spinreact, Girona, Spain) as previously described [35].

### 2.7. Western Blot Analysis

Western blot was performed as previously described [36, 37]. White adipose tissue (WAT) and brown adipose tissue (BAT) were dissected and stored at −80°C until further processing.

WAT and BAT were homogenized in ice-cold lysis buffer containing 50 mmol/L Tris-HCl (pH 7.5), 1 mmol/L EGTA, 1 mmol/L EDTA, 1% Triton X-100, 1 mmol/L sodium orthovanadate, 50 mmol/L sodium fluoride, 5 mmol/L sodium pyrophosphate, 0.27 mol/L sucrose, 0.1% 2-mercaptoethanol, and complete protease and phosphatase inhibitor cocktail (1 tablet/50 mL; Roche Diagnostics, Mannheim, Germany). Homogenates were centrifuged at 13,000 g for 10 min at 4°C, supernatants were removed, and aliquots were stored in −80°C.

WAT and BAT lysates were subjected to SDS-PAGE gels. Briefly, total protein lysates from WAT and BAT (20 *μ*g) were subjected on 8% and 12% of SDS polyacrylamide gels and electrotransferred on a polyvinylidene difluoride membrane. Membranes were blocked for 1 h in TBS-Tween 20 (TBST: 50 mmol/L Tris-HCl [pH 7.5], 0.15 mol/L NaCl, and 0.1% Tween 20) containing 3% of BSA and probed with for 16 h at 4°C in TBST, 3% BSA in the indicated antibodies: IL-6 (ab-6672), IL-1*β* (ab-9722) (Abcam, Cambridge, UK); PGC1*α* (sc-13067) (Santa Cruz Biotechnology, Santa Cruz, CA), *α*-tubulin (T-5168), and *β*-actin (A-5316) (Sigma-Aldrich). For protein detection we used horseradish peroxidase-conjugated secondary antibodies (Daku Denmark, Glostrup, Denmark) and chemiluminescence (Pierce ECL Western Blotting Substrate, Thermo scientific, USA). Then, the membranes were exposed to X-ray film (Super RX, Fuji Medical X-Ray Film, Fujifilm, Japan) and developed with developer and fixing liquids (AGFA, Germany) under appropriate dark room conditions. We used seven samples per group and the protein levels were normalized to *β*-actin (WAT) and to *α*-tubulin (BAT) for each sample.

### 2.8. Statistics

The results are shown as the mean ± standard error of mean. Statistical analysis was performed using Student's *t*-test (when two groups were analysed) or one-way ANOVA followed by a* post hoc* multiple comparison test (Tukey Test) (when more than two groups were analysed). A *P* value less than 0.05 was considered statistically significant; Graph Prism software (San Diego, CA) was used for the data analysis. For correlation studies statistical analyses were performed using SPSS version 20.0 software statistical package (SPSS, Chicago, IL). The relationships between variables were analyzed by Pearson's correlation (normally distributed data) or Spearman's rank correlation (nonnormally distributed data) coefficients (*r*). A *P* value less than 0.05 was considered statistically significant.

## 3. Results

**Figure 1:**
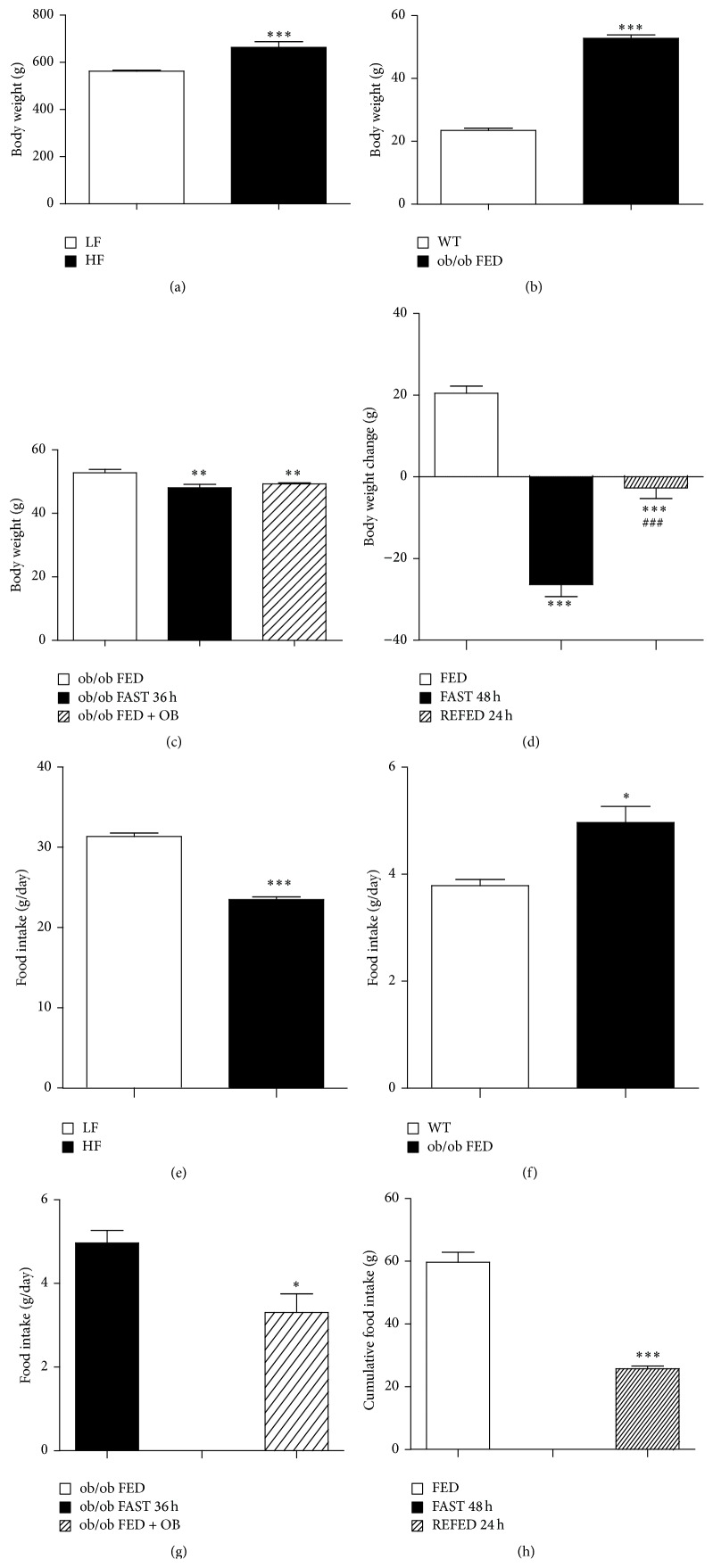
Body weight and food intake. (a) Body weight of rats fed with high fat (HF) and low fat (LF) diet. (b) Body weight in leptin deficient mice (ob/ob) versus wild type (WT) mice fed* ad libitum*. (c) Body weight in ob/ob mice fed* ad libitum*, 36 hours fasted and after leptin treatment in ob/ob mice fed* ad libitum*. (d) Body weight change in rats fed* ad libitum*, in 48 h of fasting and in rats submitted to a refeeding for 24 h after 48 h of fasting. (e) Food intake of rats fed with high fat (HF) and low fat (LF) diet. (f) Food intake in leptin deficient mice (ob/ob) versus wild type (WT) mice fed* ad libitum.* (g) Food intake in ob/ob mice fed* ad libitum*, 36 hours fasted and after leptin treatment in ob/ob mice fed* ad libitum*. (h) Food intake in rats fed* ad libitum*, in 48 h of fasting and in rats submitted to a refeeding for 24 h after 48 h of fasting. Values are mean ± standard error of the mean of 6–12 animals per group. Values are mean ± SEM. ^*∗*^ *P* < 0.05; ^*∗∗*^ *P* < 0.01; ^*∗∗∗*^ *P* < 0.001 versus controls and ^#^ *P* < 0.05; ^##^ *P* < 0.01; ^###^ *P* < 0.001 versus FAST 48 h.

**Figure 2:**
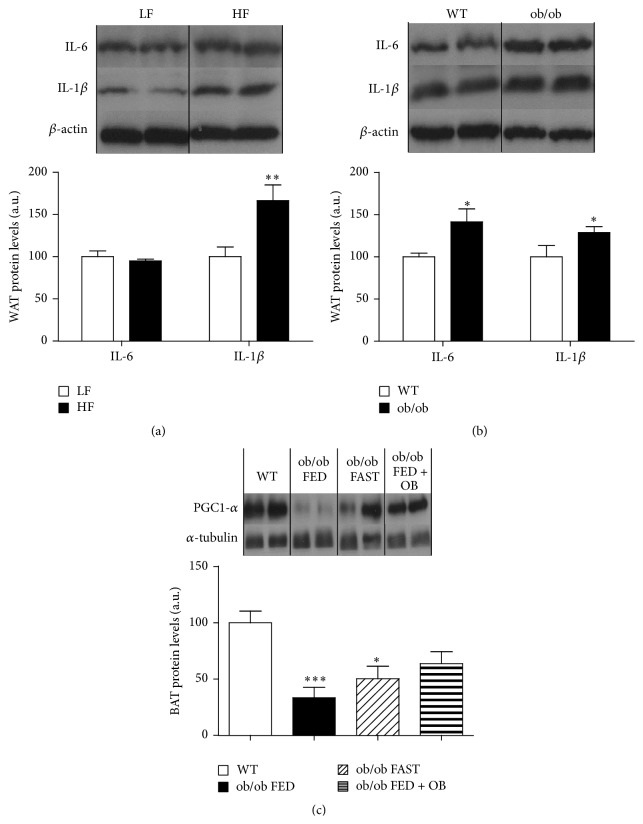
Measurements of protein levels in white and brown adipose tissue. WAT protein levels of IL-6 and IL-1*β* in DIO animals (a) and in ob/ob mice (b). BAT protein levels of PGC1-*α* in ob/ob mice (c). *β*-actin for WAT and *α*-tubulin for BAT were used to normalize protein levels. Values are mean ± SEM of 7-8 animals per group. ^*∗*^ *P* < 0.05; ^*∗∗*^ *P* < 0.01; ^*∗∗∗*^ *P* < 0.001 versus controls.

As expected, the higher body weight in rats fed HF diet (Figure 1(a)) was consistent with a significant increase in fat and lean mass index compared to LF diet fed rats (Table 1) and a significant increase in proteins levels of IL-1*β* in WAT but not of IL-6 (Figure 2(a)). We do not have a clear explanation for this fact but it could be due to the limited time of exposition to the diet (10 weeks) or because a total positive correlation between IL-6 and obesity is not always a strong proof, due to the fact that a lack of IL-6 has been shown to cause obesity and insulin resistance in rodents [38]. However, the DIO rats eat less amount of food than the LF diet fed rats because the HF diet is a hypercaloric food and these rats need less quantity of food to be satiated compared to hypocaloric LF diet fed animals (Figure 1(e)). Correspondingly, these DIO rats are hyperglycemic and hyperinsulinemic (Table 1) and display a trend of high cholesterol levels although this trend is not significant (Table 1), as previously described [22]. With regard to the monogenic model of obesity, the ob/ob mice exhibit a high body weight (Figure 1(b)) and food intake (Figure 1(f)), and similar to the DIO rats, the ob/ob mice display a significant increase in insulin, glucose, and cholesterol levels with respect to WT controls (Table 1) and high levels of inflammatory markers in WAT (Figure 2(b)). Furthermore, we measure the levels of these biochemical and hormonal parameters in the serum of rats submitted to short-term changes in nutritional status. As expected, a 48 h fasting induces a significant decrease in glucose and insulin serum levels compared to* ad libitum* fed animals and a significant increase in total cholesterol levels (Table 1) [39, 40].

**Figure 3:**
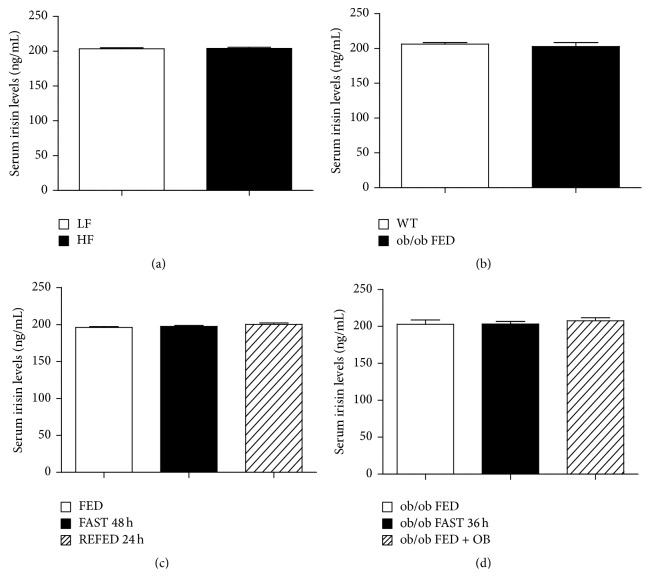
Serum irisin levels. (a) Serum irisin levels in rats fed with high fat (HF) and low fat (LF) diet for 10 weeks (b) Serum irisin levels in leptin deficient mice (ob/ob) versus wild type (WT) mice fed* ad libitum*. (c) Serum irisin levels in animals fed* ad libitum* (FED), fasted for 48 hours (FAST 48 h) or refed for 24 hours (REFED 24 h) after 48 hours of fasting. (d) Serum irisin levels in ob/ob mice fed* ad libitum*, 36 hours fasted, and in ob/ob mice fed* ad libitum* after leptin treatment. Values are mean ± SEM of the mean of 6–12 animals per group. A *P* < 0.05 was considered significant.

Since circulating irisin levels are increased in human obesity [19, 20], we assessed serum irisin levels in two animal obese models: DIO rats as a model of polygenic obesity and ob/ob mice as a model of monogenic obesity. In both rodent obese models, serum irisin levels remained unaltered. Rats fed a HF diet showed similar serum irisin levels as animals fed a LF diet during 10 weeks (Figure 3(a)). Interestingly, these results are consistent with a recent study in humans [22]. Similarly, circulating irisin levels remained unaltered between WT and obese mice lacking leptin (ob/ob mice) (Figure 3(b)). Of note, lack of differences in circulating irisin in both models of obesity was found in spite of different body weights (Figures 1(a) and 1(b)). As irisin levels were unchanged in animal models of long-term obesity, we next aimed to determine whether irisin might be regulated by short-term changes in nutritional status. In order to test this hypothesis, we measured irisin in animals fed* ad libitum* and after food deprivation. Serum irisin levels remained unaltered after 48 h of fasting or when rats were refed for 24 h (Figure 3(c)). As leptin is one of the main players in the regulation of energy balance, we carried out a food deprivation and leptin replacement in ob/ob mice to corroborate our previous data. Additionally, we measured the levels of PGC1-*α*, a proposed upstream regulator of irisin in BAT. We found a very pronounced decreased in protein levels of PGC1-*α* in ob/ob mice compared to WT mice (Figure 2(c)); however, these levels were increased by leptin treatment (Figure 2(c)) as previously described [41, 42].

In agreement with our previous findings, we failed to detect significant changes in circulating irisin levels in ob/ob mice fasted for 36 h in comparison to ob/ob mice fed* ad libitum* (Figure 3(d)), and the administration of exogenous leptin to ob/ob mice did not cause any significant alteration in serum irisin levels (Figure 3(d)). However, even though the food deprivation and leptin caused a marked decrease in the body weight (Figure 1(c)) and food intake (Figure 1(g)) and changes in proteins levels of PGC1-*α* (Figure 2(c)) of leptin-deficient mice.

Of note, this lack of differences in serum irisin levels in all of these animal models was replicated in an independent experiment by using a Radioimmunoassay (data not shown).

**Figure 4:**
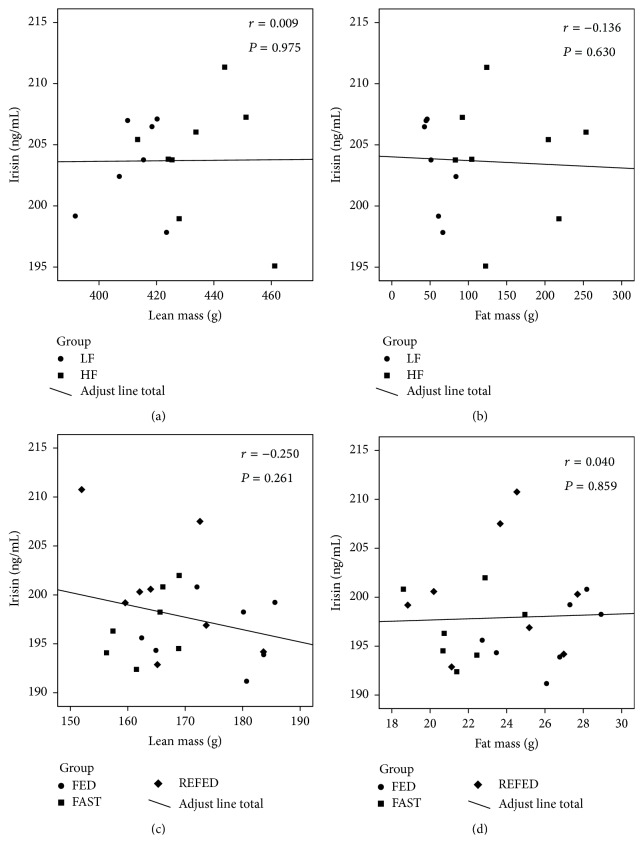
Correlation of irisin levels with body composition parameters. Correlation of irisin with lean mass (a) and fat mass (b) in DIO animals and with lean mass (c) and fat mass (d) in short-term changes of nutritional status.

**Figure 5:**
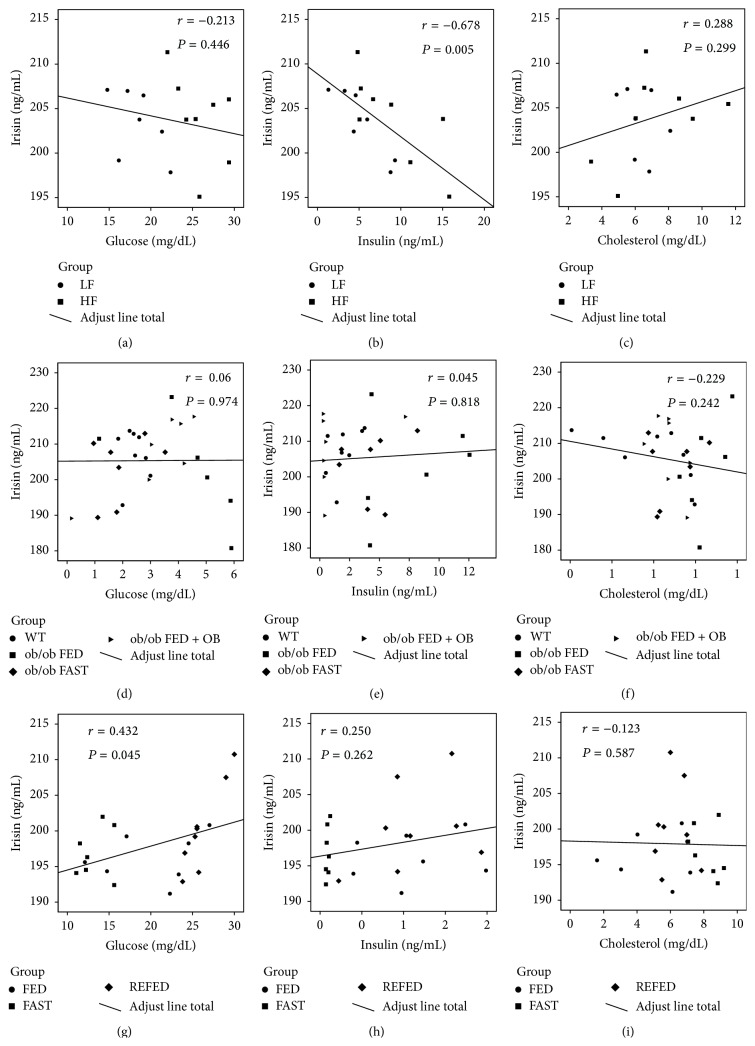
Correlation of irisin levels with biochemical and hormonal parameters. Correlation of irisin with glucose (a), insulin (b), and cholesterol (c) in DIO animals; with glucose (d), insulin (e), and cholesterol (f) in ob/ob mice; with glucose (g), insulin (h), and cholesterol (i) under short-term changes of nutritional status.

Finally, we have also performed correlations studies between serum irisin versus body composition parameters and biochemical and hormonal levels (Figures 4 and 5; Table 2). We do not find any correlation between fat mass, lean mass, or cholesterol levels and irisin in the different models studied (Figures 4(a), 4(b), 4(c), and 4(d); Figures 5(a), 5(c), 5(d), 5(e), 5(f), 5(h), and 5(i); Table 2); however, we detect a negative correlation between irisin and insulin in the DIO model (Figure 5(b); Table 2) as previously described in humans [21] and a positive correlation between glucose and irisin under short-term changes in nutritional status (Figure 5(g); Table 2).

## 4. Discussion

The discovery of irisin has created a great expectation due to its proposed beneficial metabolic effects. However, the knowledge about irisin regulation and secretion is still scant and the results are controversial. In this sense, the levels of circulating irisin in obesity have been reported to be negatively associated to obese men [21], whereas others indicated that irisin levels are high in obese individuals or are positively correlated to body mass index [19, 20, 23, 43]. In rodents, the results are equally ambiguous, as it was shown that serum irisin levels are decreased in obese Otsuka Long-Evans Tokushima Fatty (OLETF) rats in comparison to lean rats [25], and in obese Zucker rats [26] but increased in DIO rats [26]. In the current study, we have analyzed serum irisin levels in two different obese animal models, DIO rats as a model of polygenic obesity and in ob/ob mice as a model of monogenic obesity. In both cases, our results were identical, as we did not detect any significant difference between lean and obese animals in concordance with a study in obese humans [22]. Although all these results obtained in different models of obese animals do not point in the same direction, it is important to highlight that each model is associated with different metabolic alterations that might affect irisin levels. For instance, OLETF rats are diabetic whereas DIO rats are insulin resistant but not diabetic. This is a key issue since the role of irisin on glucose metabolism is still under debate [21, 32, 44, 45]. Another crucial aspect that requires attention is the methodology used for the measurement of irisin. These methodological aspects are likely affecting the results in DIO rats. In this sense, the study of Roca-Rivada and colleagues assessed serum irisin levels by Western blot [26] whereas we used a radioimmunoassay-based method. In spite of these discrepancies between different animal models or methodologies, our present results suggest that circulating irisin levels are not affected by obesity in rodents.

Since obese rodents represent models with long-term metabolic alterations and compensatory mechanisms, we next evaluated if irisin levels might be affected in situations of metabolic alterations at short term. The nutritional status induces changes in a variety of endocrine axis and if irisin has been proposed as a metabolic regulator, we hypothesized that it should be regulated by fasting. However, we failed to find any change in serum irisin levels by caloric restriction or refeeding, indicating that its circulating levels are not affected by nutritional status. Our findings are in agreement with a previous study indicating that weight loss induced by caloric restriction did not regulate circulating irisin levels in rats [46]. On the other hand both fasting and caloric restrictions are associated to a number of metabolic alterations, and one of the most relevant is the decreased leptin levels. Previous works have suggested a possible cross talk between leptin and irisin because irisin levels are associated with leptin in humans and rats [21, 25], and leptin increases mRNA expression of PGC1-*α* [41, 42], the proposed upstream regulator of irisin [8]. Moreover recently it was shown that leptin treatment slightly upregulates circulating irisin levels in ob/ob mice [47]. Therefore, we decided to investigate in depth the possible specific interaction between leptin and irisin. In order to test this interaction, we measured irisin levels in a model of hypoleptinemia performing a leptin replacement in ob/ob mice. Our data indicate that circulating levels of irisin remained unaltered after leptin administration in those mice in any experimental group. Therefore, these results suggest that serum irisin levels are not modulated by leptin on our experimental paradigms. Finally, we perform correlation studies of irisin with body composition, biochemical and hormonal parameters. In concordance with our previous results we do not find any correlation between them in most of the parameters studied (Figures 4(a), 4(b), 4(c), and 4(d); Figures 5(a), 5(c), 5(d), 5(e), 5(f), 5(h), and 5(i); Table 2); however, in spite of these negative results we detect a negative correlation between irisin and insulin in DIO animals as previously described in obese subjects [21] and a positive correlation between irisin and glucose under short-term changes in nutritional status (Figures 5(b) and 5(g); Table 2). These data indicate that irisin could play a role in states that involve impairments of glucose homeostasis.

In summary, we conclude that serum irisin levels seems in light of our present results not affected by obesity, nutritional status, or leptin in rodents. However, it is important to note that a limitation of the interpretation of the present results is that the data are based on a direct determination of irisin on serum samples without mechanism exploration or signal transduction studies of irisin with metabolism in obesity models. Further research on irisin will be necessary to clarify its precise role in the regulation of energy balance and its potential therapeutic use in obesity and its comorbidities.

## Figures and Tables

**Table 1:** Body composition and biochemical and hormonal characteristics from the models studied.

Group	Insulin (ng/mL)	Glucose (mg/dL)	Total cholesterol (mg/dL)	Fat mass (g)	Lean mass (g)
Low fat	5.36 ± 1.08	18.51 ± 1.02	6.32 ± 0.40	56.53 ± 5.63	412.3 ± 4.0
High fat	9.07 ± 1.58^*∗*^	25.86 ± 0.96^*∗∗∗*^	7.14 ± 0.92	150.02 ± 23.04^*∗∗*^	435.1 ± 5.5^*∗∗*^

Wild type mice	2.01 ± 0.42	2.41 ± 0.13	0.78 ± 0.07	—	—
ob/ob mice FED	7.67 ± 1.63^*∗∗*^	4.40 ± 0.72^*∗*^	1.04 ± 0.03^*∗*^	—	—
ob/ob mice FAST	4.35 ± 0.85	1.94 ± 0.34^##^	0.088 ± 0.04	—	—
ob/ob mice FED + OB	1.32 ± 0.97^###^	3.24 ± 0.56	0.087 ± 0.02	—	—

Rats FED	1.11 ± 0.22	20.16 ± 2.09	5.09 ± 0.83	26.21 ± 0.88	175.6 ± 3.4
Rats FAST	0.09 ± 0.007^*∗∗*^	13.23 ± 0.71^*∗∗*^	8.20 ± 0.32^*∗∗*^	21.94 ± 0.71^*∗*^	163.5 ± 1.9^*∗*^
Rats REFED	1.13 ± 0.19^##^	26.12 ± 0.78^*∗*/###^	6.14 ± 0.34^#^	23.52 ± 1.13	166.6 ± 3.4

Values are mean ± SEM of 7-8 animals per group. ^*∗*^ *P* < 0.05; ^*∗∗*^ *P* < 0.01; ^*∗∗∗*^ *P* < 0.001 versus controls. ^#^ *P* < 0.05; ^##^ *P* < 0.01; ^###^ *P* < 0.001 versus ob/ob FED and versus FAST 48 h.

**Table 2:** Correlations of irisin with body composition and biochemical and hormonal parameters from the models studied.

Group	Irisin				
	Insulin (ng/mL)	Glucose (mg/dL)	Total cholesterol (mg/dL)	Fat mass (g)	Lean mass (g)
DIO	*r* = −0,668	*r* = −0,213	*r* = 0,288	*r* = −0,213	*r* = −0,213
	*P* = 0.005^*∗∗*^	*P* = 0.446	*P* = 0.299	*P* = 0.446	*P* = 0.446

ob/ob mice	*r* = 0,045	*r* = 0,06	*r* = −0,229	—	—
	*P* = 0.818	*P* = 0.974	*P* = 0.242	—	—

FED-FAST-REFED	*r* = 0,250	*r* = 0,432	*r* = −0,123	*r* = −0,250	*r* = 0,040
	*P* = 0.262	*P* = 0.045^*∗*^	*P* = 0.587	*P* = 0.261	*P* = 0.859

Statistical significance is from Pearson (normally distributed data) and from Spearman (nonnormally distributed data) correlation test.
